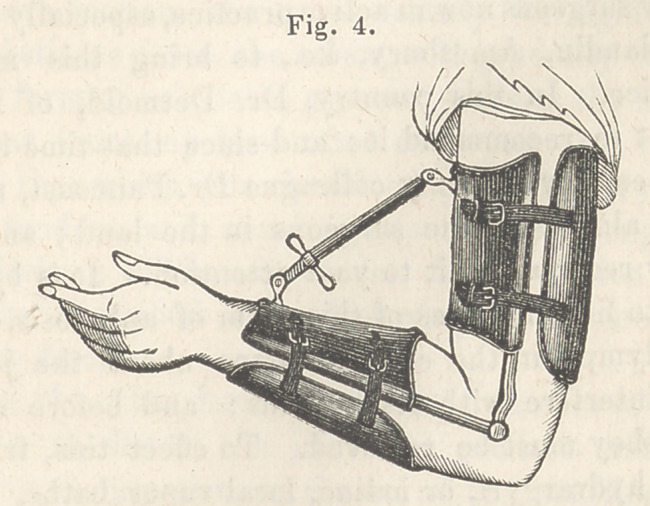# Clinic of Jefferson Medical College

**Published:** 1851-02

**Authors:** C. W. Hornor

**Affiliations:** Senior Clerk of the Surgical Department


*Clinic of Jefferson Medical College. Service of* PROFS. MÜTTER and PANCOAST. Report furnished by C. W. Hornor, M. D., Senior Clerk of the Surgical Department.

### TRUE ANKYLOSIS OF KNEE.

(Concluded.)

*Treatment.*—From what I have already told you, it must be obvious that the treatment will vary essentially in our attempts to cure ankylosis. In the true or complete form of the affection, where the union is cartilaginous, bony, or composed of short and dense bands of fibro-cartilaginous, or ligamentous tissue, attempts have been made to establish motion by the application of a machine of sufficient power to break up, either suddenly, or by degrees, the cause of the defect; but invariably have these attempts resulted in a failure to accomplish the rupture of the bond of union, or where this has been effected, in acute inflammation of the part with all its usual effects, and sometimes in the death of the patient. Louvrier, of Paris, is the last of those who advocate this plan of treatment, (which had long before this date been practised by Lafond, Hildanus, and others, in false ankylosis,) but the unfavorable report of the "Academy" relative to his success, will be sufficient to consign his measure "to the tomb of all the Capulets."

In striking contrast with this operation of Louvrier, are those for the same affection introduced by Dr. J. Rhea Barton, of Philadelphia. I do not hesitate to assert, that the age has given birth to nothing more brilliant, more profoundly philosophical, more eminently useful, or better calculated to shed lustre upon our science. Two plans of relieving the deformity from stiff joints have been proposed by Dr. Barton. In one, the establishment of an *artificial joint* is the object in view. In the other, *the removal of a portion of one or more of the bones involved, by which a limb flexed at any angle may be made straight.* That a false joint might be established by first cutting through a bone, and then keeping up motion between its divided ends, was a point fully established by the experiments of different surgeons, especially Chaussier, Köcler, Sir A. Cooper, and Larrey, and also by the success which had followed the excision of joints, in cases of caries, &c., by Park, Moreau, and others; but Dr. Barton was the first to propose the application of the principle in the treatment of ankylosis. The manner of performing the operation will, of course, vary in each case; in one it may be proper to carry the incision through the original joint; in another through the bone immediately above or below it. In the first case of Dr. Barton's, that of a sailor, in whom the hip joint had been injured by a fall, and which was characterized by great deformity, the latter operation was performed, the femur being sawed through "at the lower part of its cervix, a little above its root." This plan should always be preferred when practicable, especially where the ankylosis has been the result of previous ulceration in the joint, inasmuch as we avoid by it the risk of exciting anew the disease in its original seat. When the operation succeeds, the false joint may resemble, to a certain degree, an original one, not only in its functions, but also in its anatomical characters. The bones, for example, are tipped with cartilage, and covered with a layer of condensed cellular tissue, which strongly resembles synovial membrane; but usually, instead of this, the bones are united to each other by ligamentous matter, so flexible that it yields to the contractions of the different muscles surrounding the joint, and thus the limb becomes subject to the will, and is almost as useful as before the destruction of its original articulation.

But although this operation of Dr. Barton's is one of the most ingenious and beautiful in surgery, it must not be performed in every case, and without deep reflection. In the first place, it is unquestionably a hazardous procedure, and subjects the patient to great danger; and in the second, it is liable to be followed by a return of the defect, bony matter being sooner or later deposited in the connecting medium. I cannot do better, however, than give you the advice of Dr. Barton himself. This operation he states, is justifiable only under the following circumstances, viz:

"When the patient's general health is good, and his constitution is sufficiently strong; when the rigidity is not confined to the soft parts, but is actually occasioned by a consolidation of the joint; where all the muscles and tendons that were essential to the ordinary movements of the former joint are sound, and not incorporated by firm adhesions with the adjacent structure; where the disease causing the deformity has entirely subsided; where the operation can be performed through the original point of motion, or so near it that the use of most of the tendons and muscles will not be lost; and finally, where the deformity or inconvenience is such as will induce the patient to endure the pain and incur the risk of an operation."

An operation similar in principle to this of Dr. Barton's has been performed, according to Professor Samuel Cooper, by Mr. Anthony White, of London.

The second method of operating proposed by Dr. Barton, is intended for the relief of those cases in which, from the size of the joint, and the shape of the limb, it would be hazardous or impossible to attempt the establishment of an artificial joint. So far it has been limited to operations upon the lower extremities; but in deformities of the upper it would be equally useful. The case operated on by Dr. Barton, a report of which you will find in the American Journal of the Medical Sciences of 1838, was one of bony ankylosis of the knee joint, attended with great angular deformity, the leg forming nearly a right angle with the thigh.

The operation consisted in first exposing the femur just above the patella by a triangular incision, the base of the triangle resting upon the front of the thigh; then removing from it by means of a small saw, a wedge-like piece, and finally, in gradually bringing the limb down, by a double inclined plane, the inclination of which could be varied at pleasure to a straight position, and retaining it there until union took place. In order to protect the popliteal artery, and also to steady the fragments by the interlocking of the asperities of each, the incisions with the saw were not carried entirely through the bone, but terminated within a few lines of its posterior surface. The solution of continuity was then rendered complete by an attempt to bend the bone, which caused, of course, the fracture of that portion which had not been divided with the saw. The operation was perfectly successful, and is precisely similar to the one you have just witnessed.

Either of these means is vastly preferable to the *excision of the joint*, advised by some, as well as to the "amputation de complaisance," recommended by certain French surgeons.

Neither excision of the joint nor amputation, two of the most dangerous operations in surgery, should be performed for the removal of what is merely an inconvenience, and my advice is this, refuse to the last any entreaty of the patient, who may urge the performance of them upon you.

In those cases of *false ankylosis* of the knee in which *subluxation of the tibia has gone so far as to forbid any attempt to relieve their defect for fear of producing complete luxation, the operation of Dr. Barton might be performed, and the case of William Blakely will, at a subsequent period, be treated in this manner.*

We come next to speak of the treatment of *false ankylosis.* And here, before undertaking the management of the case, or giving our prognosis, it is absolutely essential for the proper remedies to be applied that a correct diagnosis should be formed. When the stiffness of a joint is evidently the result of *rest,* no previous disease of its various constituents having occurred, the difficulty may generally be removed by passive motion, frictions with oleaginous substances, electro-magnetism or galvanism, the vapor bath, fomentations of various kinds, hot mineral baths, especially those of Virginia, and finally, in bad cases, by the use of an instrument similar to that applied in the case before you. When the limb is flexed, the screw must be worked so as to separate one part from the other; when it is straight, the motions of the screw are reversed, so as to approximate them.

Fig. 4.

The disgusting practice of enveloping the part in the hot entrails of a recently slaughtered animal, recommended by Boyer and others,

I need hardly tell you, should not be resorted to, inasmuch as it is productive of nó benefit that cannot be obtained by less revolting remedies. (See fig. 4, for a representation of the "screw" applied in a case of false ankylosis of the elbow.) When the joint is rendered motionless by the contraction of the skin after a burn, an ulcer, or an abscess, the treatment must be based upon the principles laid down when I called your attention to the subject of cicatrices; usually, but not universally, a plastic operation is required.

When previous inflammation of the extra-articular tissues, has given rise to stiffness, we must be exceedingly careful in our attempts to give relief, or we may cause the disease to reappear. In all such cases I employ the screw of Stromeyer, along with the remedies usually resorted to in simple stiffness from rest; and there can never be a necessity here for the knife. Should the instrument cause pain or inflammation in the joint, I at once suspend its extending action, and merely employ it as I would a carved splint, to keep the part at rest, while at the same time I order leeches, cold applications, low diet, purging, &c., and never renew extension or flexion, as the case may be, until all traces of inflammatory action have disappeared. The practice of treating such cases by an extending process is by no means a novel procedure; for we find that Hildanus, Lafond, Boyer, and others, employed machines very similar to those made use of by us at the present time. It is true, however, that much has been done by surgeons now in active practice, especially Stromeyer, Lisfranc, Blandin, Amesbury, &c., to bring this method into general notice. In this country, Dr. Detmold, of New York, was the first to recommend it; and since that time it has been extensively employed by my colleague Dr. Pancoast, myself, Dr. Chase, and almost all the surgeons in the land; and I cannot too strongly recommend it to your attention. It is by no means uncommon to find, in cases of this form of ankylosis, deposits of coagulable lymph in the cellular tissue about the joint, which materially interfere with its motions; and before a cure can take place they must be removed. To effect this, friction with unguentum hydrargyri, or iodine, local vapor baths, but, above all, pressure with adhesive straps and a bandage, should be at once employed.

When the ankylosis is dependent upon a contraction of fascia, as we see in certain deformities of the knee, ankle, sole of the foot, elbow, palm of the hand and fingers, although mechanical means may answer, still it is necessary to resort to the knife. My own practice in these cases is, to make the attempt first by mechanical measures alone—the apparatus being modified to suit the case; and should these fail to acomplish the object in three or four weeks, I then divide the fascia. The operation is very simple, and is performed with a small scalpel, which is introduced between the skin and the fascia, just as in the operation for clubfoot, and then turning it upon its edge, it is made to incise the resisting tissue from without inwards. The knife is then withdrawn, the little puncture made in the integuments closed with adhesive plaster, and the extending apparatus applied. Gradual extension may be at once commenced, but we must carefully avoid being in too great haste to effect a cure, for fear of exciting, by our efforts, inflammation. It is also highly important to commence *passive motion* in the course of a few days after the extension is completed, and to *keep up extension* for some weeks after the limb has assumed its natural shape ; unless attention is paid to these two points, the deformity will almost to a certainty reappear.

Stiffness occasioned by contractions of the muscles and tendons, the result of rest, paralysis of antagonists, and sloughing, require to be treated with much discrimination. When the contraction is organic, and may be traced to rest of the joint too long continued, passive motion, the usual remedy for rigid joints, and lastly, in obstinate cases, the screw, will generally effect a cure. It is in this form of ankylosis, especially where the knee joint is involved, that tenotomy is so often employed ; but the practice indicated is that which you should adopt. First try the screw; should the tendons and muscles resist for any length of time, then their division will be admissible. The operation is precisely similar to that described as the best when it becomes necessary to divide the fascia.

When, on the other hand, paralysis of one set of muscles allows another to distort the joint and produce ankylosis, it is impossible by an operation, or the use of any machine, to restore the part to its normal condition. In such cases we sometimes derive benefit from the application of the remedies supposed to exert a favorable influence in palsy, such as galvanism, electricity, frictions with veratria and strychnia, cold bathing, &c. In recent cases I have sometimes found advantage from the application of a splint, which prevented the distortion of the limb, while the remedies for the paralysed muscles were being administered. I have also tried in one case of palsy of the extensors of the hand, a contrivance recommended by Dr. Charles Bell, and by its use prevented ankylosis, and enabled the patient to employ the member in his ordinary avocations. This machine was composed of four pieces of steel, of sufficient power to keep the fingers straight, when no effort was made to flex them, but not strong enough to resist the voluntary action of the flexors, placed along each finger, and fastened at one extremity into a bracelet around the wrist, and at the other into a common brass thimble. The fingers being inserted into the thimbles, and the bracelet fastened around the wrist, the springs took the place of the extensor tendons, and a glove being drawn over the whole, it was impossible to detect the appearance of anything unnatural.

When sloughing, or destruction of the tendons or muscles by a wound, is the cause of the ankylosis, it is of course impossible to accomplish a cure. The same may be said of those cases in which the tendons are bound down very firmly by adhesions, the result of previous acute inflammation. When gout, rheumatism, sprains, luxations, synovitis, or disease of the cartilage or bones have given rise to capsular or intra-capsular ankylosis, the treatment is very similar to that already indicated, but we must expect a much more tedious convalescence. The use in these cases, of alkaline baths, is highly recommended by certain of the French surgeons.

Finally, we should always inform the patient, that usually, in false ankylosis, it is necessary to support the limb by mechanical means for some weeks or months after it has attained its proper shape, or until the weakened muscles, fascia and tendons have regained their original tone, or at least are strong enough to prevent subsequent contraction of the part. During this period, we should employ all the best remedies for giving tone and vigor to the weakened parts, such as frictions, cold bathing, electricity, &c.; and in many cases constitutional remedies are highly important.

## Figures and Tables

**Fig. 4. f1:**